# The effects of magnesium-containing coatings on the healing of soft tissues surrounding oral titanium abutments: a narrative review

**DOI:** 10.3389/fdmed.2025.1638027

**Published:** 2025-09-25

**Authors:** Hongya Zheng, Chenxi Shang, Ping Li, Wenjie Zhao, Yumei Niu, Shuang Pan, Shuang Zhang

**Affiliations:** ^1^ Department of Endodontics, The First Affiliated Hospital of Harbin Medical University, Harbin, China; ^2^ Department of Endodontics, School of Stomatology, Harbin Medical University, Harbin, China; ^3^ NHC Key Laboratory of Cell Transplantation, The First Affiliated Hospital of Harbin Medical University, Harbin, China

**Keywords:** magnesium-containing coating, magnesium, dental implants, soft tissue healing, abutments

## Abstract

The inherent limitations of smooth titanium abutments—particularly inadequate soft tissue sealing that predisposes implants to infection and inflammation—underscore the need for surface modifications. This review synthesizes evidence on magnesium-containing coatings as a bioactive solution to enhance peri-implant soft tissue healing. Through 1) modifying the surface properties of the implant abutment to promote better cell adhesion and proliferation; 2) releasing Mg^2+^ to promote fibroblast migration, collagen synthesis, and angiogenesis; and 3) exerting antimicrobial effects and regulating inflammatory responses, these coatings establish a microenvironment conducive to robust tissue integration. This helps prevent peri-implant infections and inflammation, strengthens soft tissue attachment, and improves the long-term stability of dental implants, providing a new direction for the development of biomedical materials.

## Graphical Abstract

**Figure:**
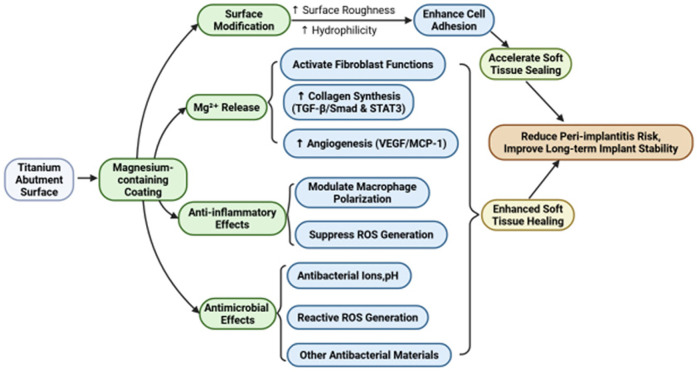

## 1 Introduction

**Figure 1:**
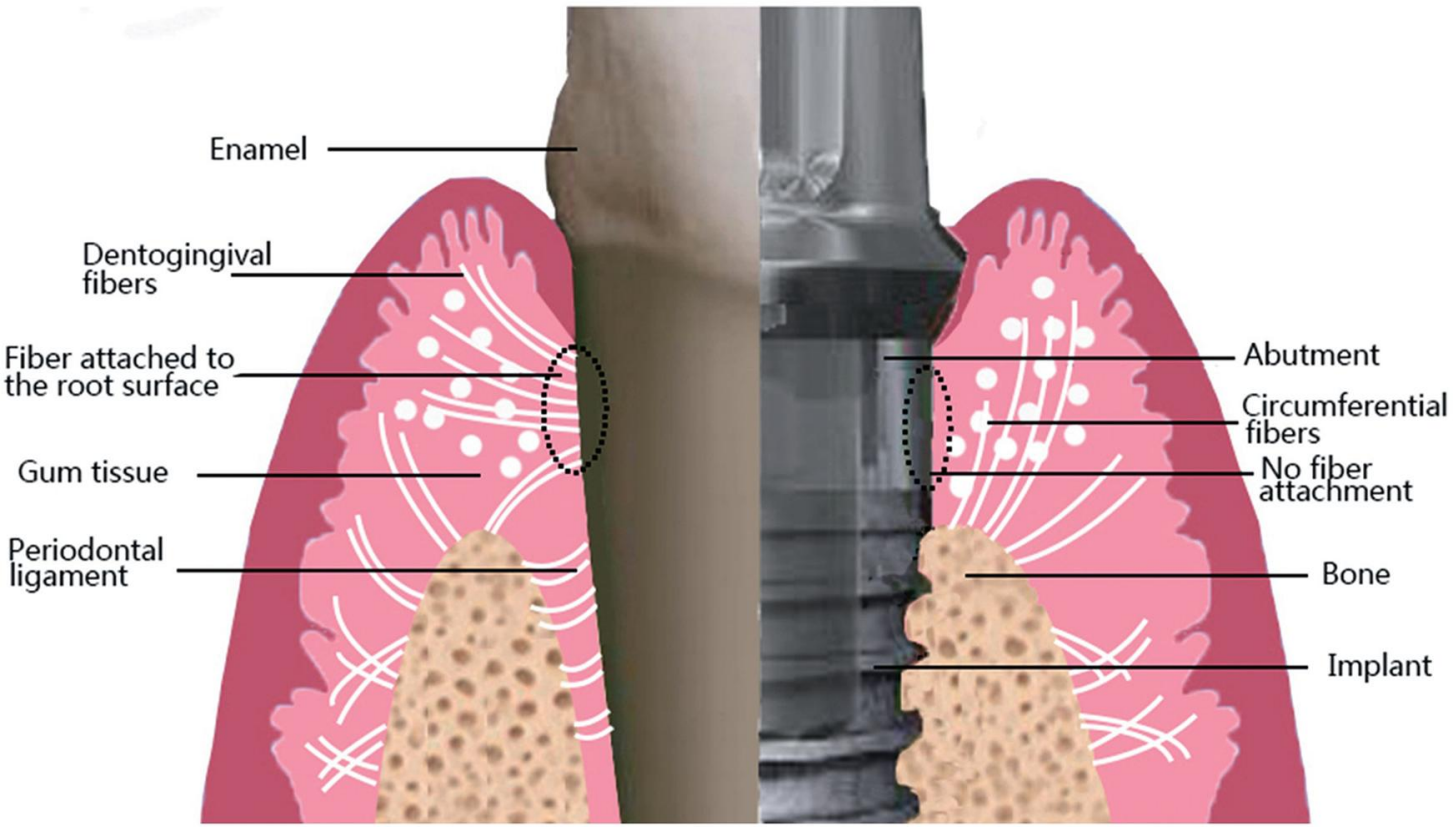
Differences between the connection of soft tissues to natural teeth and dental implants. Note the arrangement of gingival fibers in a parallel orientation on implant surfaces. This figure was adapted from Ref. (2) with permission.

Dental implants, since their advent in the 1960s, have become the primary solution for addressing tooth loss due to their efficiency, durability, and aesthetic benefits. Dental implants usually consist of three parts: an implant, the abutment, and the crown. The abutment is mainly in contact with the surrounding soft tissue. Critical to dental implant success is the peri-implant soft tissue barrier, formed by keratinized epithelium adherent to the abutment. This barrier effectively prevents bacteria and food debris in the mouth from entering the space between the implant and bone tissue, which can reduce the risk of inflammation and infection (1). However, the connective tissue around the implant is collagen fibers oriented parallel to the abutment; there are no fibers inserted into the abutment surface, which is different from the natural tooth, as shown in Figure 1 (2). As such, the soft tissue around the implant has low attachment strength, which facilitates peri-implant infection. Research has shown that the average prevalence of peri-implant inflammation in implant patients is 19.53% (3). Progressive chronic inflammation will eventually cause the implant to loosen and fall out. This fundamental biological limitation underscores the need for strategies to enhance soft tissue integration.

Magnesium (Mg), the fourth most abundant element in humans, serves as a critical cofactor in cellular metabolism, membrane stability, signaling pathways, and so on (4). Mg-containing materials have been studied for various soft-tissue repair applications, such as skin wound healing (5) and periodontal tissue regeneration (6). Mg-containing coatings gradually degrade *in vivo* to release Mg^2+^. Mg^2+^ supports cell proliferation and migration and modulates intercellular signaling, which is conductive to tissue regeneration (7). Additionally, Mg^2+^ release has been found to promote angiogenesis; this process ensures the delivery of essential nutrients and oxygen to healing tissue by enhancing the local blood supply, which can accelerate the healing process (8). Mg-containing coatings also exhibit excellent anti-inflammatory properties (9). Finally, Mg^2+^ regulates the immune response, reducing excessive inflammation to provide a more conducive environment for soft tissue healing.

Most titanium abutments are typically designed with a smooth surface. However, having a smooth surface alone is insufficient to establish a robust soft tissue seal (10). Mg-containing coatings offer a bioactive alternative by way of their unique surface properties and the release of therapeutic ions. This review synthesizes mechanistic and clinical evidence on how Mg-containing coatings enhance peri-implant soft tissue healing. We focus on their multifaceted impacts—from cellular interactions to antibacterial effects—and discuss unresolved translational barriers.

## 2 Physiological process and influencing factors of peri-implant soft tissue healing

The healing of peri-implant soft tissue is a complex and delicate physiological process. This physiological process and its main influencing factors are outlined below.

### 2.1 Physiological stages of soft tissue healing around implants

#### 2.1.1 Initial blood clot formation

After implantation, blood clots quickly form around the wound (11). These clots provide a temporary matrix for cell migration and release a variety of growth factors, such as platelet-derived growth factor and transforming growth factor–β [TGF-β]), which regulate subsequent cellular responses (12).

#### 2.1.2 Inflammatory phase

Macrophages and neutrophils are recruited to the wound site to remove damaged tissue and bacteria (13). This phase is typically accompanied by the onset of localized inflammation, which plays a role in coordinating tissue repair processes.

#### 2.1.3 Proliferative phase

Under the stimulation of growth factors, fibroblasts, endothelial cells, and epithelial cells proliferate and migrate to the wound site. During this phase, fibroblasts synthesize extracellular matrix components, such as collagen, providing structural support for the regenerating tissue (14).

#### 2.1.4 Remodeling phase

Newly formed soft tissue undergoes maturation, with collagen fibers reorganizing to enhance tissue strength and functionality (15). The result is a stable soft tissue seal that protects the implant from external invasion.

### 2.2 Factors influencing peri-implant soft tissue healing

The healing of peri-implant soft tissue is influenced by multiple factors. Abutment materials [such as titanium, zirconia (16)], surface features of abutments [such as macro design, morphology (17)], loading protocols [such as immediate implantation and early or delayed implantation (18)], host conditions [such as diabetes, smoking, immune suppression, etc (19, 20).], and microbial factors all have different effects on the soft tissue. The following will analyze factors related to the Mg-containing coating.

#### 2.2.1 Material factors

Surface characteristics of implant abutments, such as roughness, hydrophilicity, and chemical composition, directly affect soft tissue healing. Rough surfaces enhance cellular adhesion and tissue integration (21), while hydrophilic surfaces attract and retain blood proteins, promoting cell adhesion and expansion (22). Moreover, surface coatings, such as Mg-containing coatings, can release bioactive ions (e.g., Mg^2+^) to modulate cell behavior, facilitating soft tissue healing (23). The biocompatibility of the material directly affects the intensity and duration of the host's inflammatory response (24). Furthermore, the formation of new blood vessels ensures the supply of oxygen and nutrients required during the healing process. Materials design should consider promoting angiogenesis to support effective healing.

#### 2.2.2 Microbial factors

After implant placement, oral microbes rapidly colonize its surface, forming a biofilm that includes bacteria and their secreted adhesive substances. This biofilm can resist both antimicrobial agents and the host immune system (25). Once established, this biofilm may become a source of chronic inflammation, potentially leading to peri-implantitis, which can compromise soft tissue integrity and lead to bone loss. Microbial colonization and infection with organisms such as *Porphyromonas gingivalis* and *Aggregatibacter actinomy-cetemcomitans* (26) can also trigger a local immune response, which can damage soft tissue and induce a stronger inflammatory response. To address microbial factors, the antimicrobial properties of materials have gradually become a focus of research.

Understanding these influencing factors and implementing appropriate design and management strategies are essential for achieving successful implant restoration. Mg-containing coatings can enhance peri-implant soft tissue healing by improving surface characteristics, releasing Mg^2+^ to promote cell proliferation, migration, and differentiation, modulating inflammatory responses, affecting fibroblast function, collagen synthesis, and angiogenesis, and providing antibacterial effects.

## 3 Mg-containing coatings modify the surface properties of abutments to promote soft tissue healing

Abutment surface characteristics play a crucial role in soft tissue healing, with surface roughness, hydrophilicity, and chemical composition directly impacting cellular adhesion, proliferation, migration, and integration. Below, we explore how Mg-containing coatings alter abutment surface characteristics to promote soft tissue healing.

### 3.1 Increased surface roughness of abutments promotes cell adhesion

Rough surfaces mimic the natural extracellular matrix environment, facilitating directed cell migration and promoting tissue integration with the abutment surface (27). This integration accelerates the healing process and enhances the mechanical strength and functionality of the new tissue. The surface characteristics may be adjusted by flame carbonization and oxygen plasma treatment (22). The results showed that moderate roughness (roughness ratio r ≈ 2) is best for cell adhesion, growth, and proliferation, while too high or too low roughness can inhibit cell adhesion.

When Mg-containing coatings are prepared with different surface treatment techniques, they can alter the nanoscale morphology of the titanium surface of the abutment to increase its roughness. A nanostructured surface provides more adhesion sites for cells, allowing cells to attach more securely to the material surface (28). This modulation is particularly important for orderly tissue regeneration and effective wound repair. Changes in surface roughness can also be sensed by integrins associated with cell adhesion, including the focal adhesion kinase (FAK) and Src family kinase pathway (29), the Mitogen-Activated Protein Kinase/Extracellular signal-Regulated Kinase (MAPK/ERK) signaling pathway (30, 31), and the Phosphatidylinositol 3-Kinase/Protein Kinase B (PI3 K/AKT) signaling pathway (32). Studies have shown that dendritic cell adhesion influenced by titanium surface roughness is regulated via the β2 integrin–FAK–AKT signaling cascade (33). This further illustrates the notion that Mg-containing coatings enhance cell adhesion by increasing surface roughness.

### 3.2 Enhanced surface hydrophilicity of abutment promotes protein adsorption

A hydrophilic surface can facilitate better fluid transport at the abutment–soft tissue interface, enhancing the protein-adsorption capacity (34). Highly wettable hydrophilic abutment surfaces form a stable and uniform liquid film upon contact with biological fluids, which improves cell proliferation and differentiation (35). Yu et al. (36) used Mg plasma immersion ion implantation and deposition technology to fabricate Mg-containing coatings on the surface of abutments. They found that this treatment improved the hydrophilicity of the surface and enhanced the proliferation, adhesion, wound healing, and extracellular matrix formation of fibroblasts. With the addition of Mg, the hydrophilicity of the abutment surface is improved (37).

Hydrophilicity alone does not significantly increase protein adsorption, but combining hydrophilicity with nanostructures can maximize protein-adsorption levels. Nanostructures increase the surface area, providing more sites for protein adsorption, which can enhance the adsorption efficiency of protein (38). Research (37) has shown that preparing Mg-containing nanocoatings on titanium implants with wet chemical treatments enhanced the protein-adsorption capacity. By increasing adsorption of proteins like fibrinogen and platelets, a continuous protein layer is formed, which is essential for subsequent cell adhesion. Overall, Mg-containing nanocoatings increase protein adsorption, creating a favorable microenvironment for cell adhesion and expansion. Rapid cell expansion helps to cover the abutment surface, accelerating the healing process.

At present, there is a lack of detailed comparative studies on the effects of Mg-containing coatings prepared by different methods on the surface properties of abutment. Controversy persists about which surface properties are the key factors affecting the healing of peri-implant soft tissue. In addition to hydrophilicity and roughness, it is important to analyze the effects of physicochemical properties such as surface tension and surface energy on soft tissue closure.

## 4 Mg^2+^ release influences fibroblast function, promoting collagen synthesis and angiogenesis

The connective tissue surrounding implants consists of collagen fibers, matrix, cells, and blood vessels. Among these, fibroblasts and blood vessels are less distributed. They form a weak, closed area of soft tissue with collagen fibers on the abutment surface. In particular, fibroblasts are the main cell type in soft tissue repair and regeneration, which are responsible for the generation and maintenance of the extracellular matrix. Therefore, improving the number and biological activity of fibroblasts is very important to promote the healing of peri-implant soft tissue.

### 4.1 Mg^2+^ promotes fibroblast proliferation, differentiation, and migration

#### 4.1.1 Mg^2+^ promotes fibroblast proliferation and adhesion

**Figure 2:**
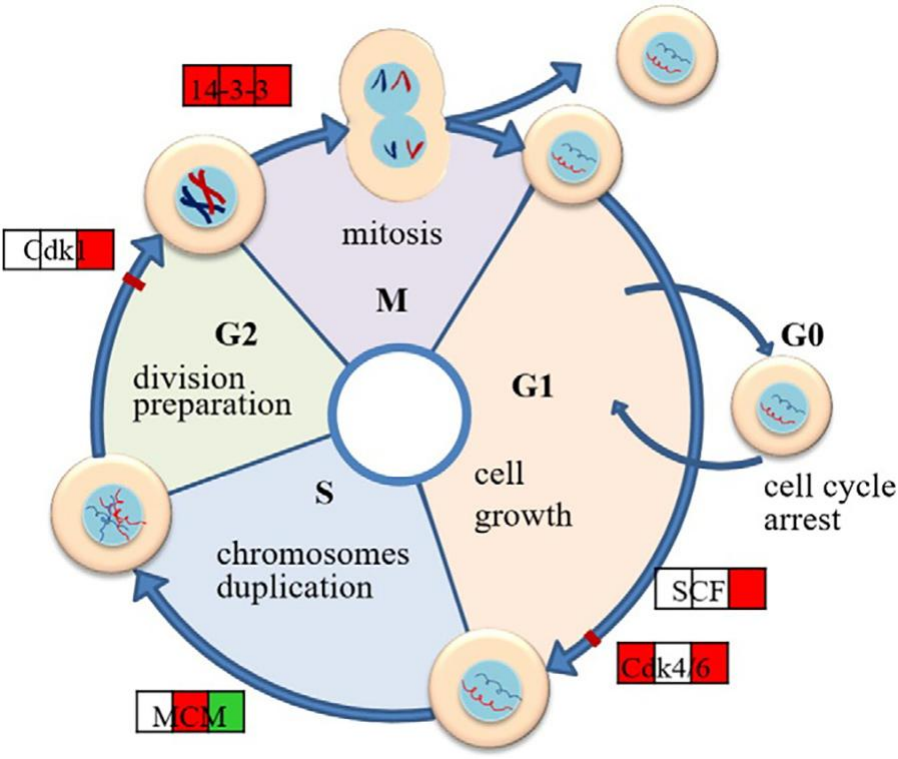
Schematic diagram of regulated cell cycle pathways in L929 exposed to Mg extract.where the three squares stand for the protein expression in 8, 24 and 48 h respectively,and red color shows that at this time point the protein is up-regulated, while green color stands for down-regulation and the blank square means no change of the expression. This figure was adapted from Ref. (40) with permission.

The trigger for cell proliferation originates from the action of growth factors. After binding growth factors to the cell surface, Mg^2+^ quickly enters the cell interior through transporters such as TRPM7 and MagT1 (39). On the one hand, Mg^2+^ is directly involved in the construction of integrins and regulates cell motility and adhesion by activating the widespread expression of adhesion molecules such as Vinculin (VCL) and FAK. On the other hand, Mg^2+^ activates signal-transduction pathways such as PI3 K/AKT or PI3 K/mTOR (Mechanistic Target of Rapamycin), thereby playing a regulatory role in cell proliferation, migration, and extracellular matrix secretion, increasing the number of fibroblasts. Zhen et al. (40) studied the effects of pure Mg extract on mouse fibroblasts by proteomic methods and found that Mg extract significantly enhanced the expression of cell cycle–related proteins, which suggests that Mg extract plays a positive role in the cell cycle pathway (Figure 2). The upregulation of key cyclin-dependent kinases (Cdks), such as Cdk1 and Cdk6, indicates that Mg^2+^ accelerates the transition from the G1 to S phase and promotes the DNA replication and division of cells, which suggests the role of Mg^2+^ in promoting fibroblast proliferation. Protein synthesis is also important for cell proliferation. Mg extracts contribute to the overall process of cellular protein synthesis by regulating multiple links such as splicing, RNA trafficking, ribosome translation, and folding and glycosylation of the endoplasmic reticulum. At the same time, Mg extract also enhances ATP production by affecting the oxidative phosphorylation process of mitochondria (40) to provide energy for cell proliferation and metabolic activities.

#### 4.1.2 Mg^2+^ promotes fibroblast migration and differentiation

Fibroblasts migrate to the wound site and begin repairing the damaged tissue. During cell migration, cytoskeletal reorganization plays a critical role, particularly the polymerization and depolymerization of filamentous actin (41). This process is associated with a Mg^2+^-dependent pathway involving RhoA, a GTP-binding protein (40). RhoA (42) regulates actin–myosin contraction by influencing myosin light chain phosphatase, facilitating stress fiber assembly and promoting cytoskeletal reorganization.Additionally, Mg^2+^ can promote fibroblast endocytosis (40). By upregulating proteins such as clathrin and adaptor protein 2, Mg^2+^ enhances the capacity of fibroblasts to uptake external signaling factors. This enables fibroblasts to respond to changes in the microenvironment during migration and to regulate cytoskeletal reorganization.

**Figure 3:**
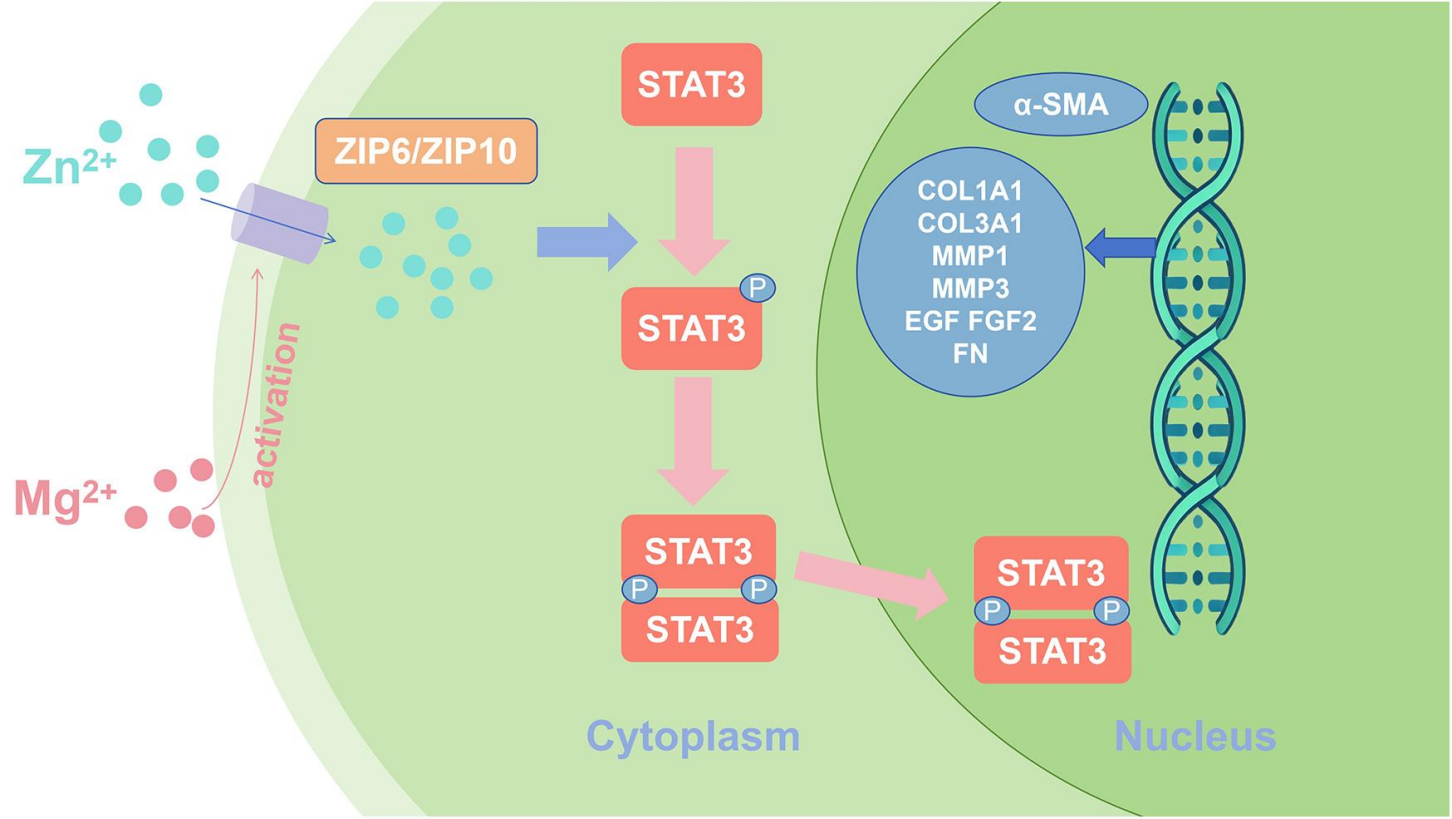
Schematic representation of magnesium and zinc ions promoting fibroblast differentiation through activation of STAT3 signaling.

Myofibroblasts play a key role in the advanced stages of wound healing, promoting wound closure and tissue reconstruction. Yang et al. (43) showed that Mg and zinc (Zn) ions can facilitate the differentiation of fibroblasts into myofibroblasts, a process achieved through activation of the STAT3 signaling pathway. Mg^2+^ upregulates the expression of ZIP6 and ZIP10 and promotes Zn^2+^ entry into fibroblasts. Increasing intracellular Zn^2+^ concentration effectively promotes STAT3 phosphorylation, which induces fibroblast differentiation into myofibroblasts and accelerates extracellular matrix deposition, as shown in Figure 3. Recent research showed that connective tissue growth factor mediates the proliferation and migration of mouse fibroblasts through the STAT3 signaling pathway (44). Myofibroblasts not only contract the wound but also synthesize proteins, including collagen and fibronectin, and remodel the extracellular matrix, thereby accelerating peri-implant soft tissue healing.

### 4.2 Mg^2+^ enhances collagen synthesis in peri-implant soft tissue

Collagen is a major component of the extracellular matrix, providing structural support in wound healing and tissue repair. The release of Mg^2+^ promotes collagen synthesis in peri-implant soft tissue through various mechanisms. On the one hand, Mg^2+^ enhances the collagen-synthesis capacity of fibroblasts by activating signaling pathways related to extracellular matrix synthesis, such as the TGF-β/Smad signaling pathway (45). On the other hand, Mg^2+^ clears old collagen fibers by activating matrix metalloproteinases (e.g., MMP-7) (46), promoting the degradation and reorganization of the extracellular matrix and creating a framework for the generation and arrangement of new fibers. Besides, Mg^2+^ regulates the recombination of actin and other cytoskeletal proteins by activating the ERK1/2 signaling pathway (46, 47). Further, it can enhance the migration and collagen-secretion capacity of fibroblasts. This helps the newly generated collagen fibers to be more orderly and closely arranged, improving the mechanical strength and functionality of the new tissue.

### 4.3 Mg^2+^ promotes angiogenesis in peri-implant soft tissue

Angiogenesis is a critical process in soft tissue repair, supplying new tissue with oxygen and nutrients. Mg^2+^ enhances the proliferation and migration of vascular endothelial cells (48), which are fundamental steps in angiogenesis. Mg^2+^ primarily interacts with G-protein–coupled receptors and calcium-sensing receptors on cell surfaces to initiate downstream signal-transduction pathways (49). Activation of these receptors raises intracellular calcium levels, which triggers a series of processes related to cytoskeletal reorganization and focal adhesion protein disassembly. This disassembly reduces cell adhesion to the matrix, making cells more “loosened” and thus more mobile. Furthermore, Mg^2+^ supports directed cell migration by increasing the expression of certain chemokines, such as monocyte chemoattractant protein–1 and interleukin (IL)-8 (36, 50). Mg^2+^ also promotes the expression of angiogenic factors, such as vascular endothelial growth factor (49), which stimulates endothelial cell proliferation and induces new blood vessel formation, both crucial to accelerate soft tissue healing.

It is worth mentioning that diabetes mellitus has been recognized as a risk factor for unsuccessful implant therapy. Liu et al. (51) established a diabetic mouse model and developed an Mg-coated implant by hydrothermal synthesis. These implants were found to successfully improve vascularization and osseointegration in the diabetic state. Mechanismally, Mg^2+^ promotes the degradation of Kelch-like ECH-associated protein 1 and the nucleation of nuclear factor erythroid 2–related factor 2 by up-regulating the expression of sestrin 2 in endothelial cells, which can reduce elevated oxidative stress levels in mitochondria and alleviate endothelial cell dysfunction under hyperglycemic conditions.

The release of Mg^2+^ significantly accelerates soft tissue repair and regeneration by promoting fibroblast proliferation, migration, differentiation, collagen synthesis, and angiogenesis. Through the activation of multiple key biological signaling pathways, Mg^2+^ not only supports extracellular matrix formation and tissue reconstruction but also ensures the long-term survival and functionality of new tissue by improving blood supply. Future studies can further optimize the release behavior of Mg-containing coatings to make more effective use of these mechanisms in clinical applications and improve the effect of soft tissue repair.

## 5 Mg-containing coatings modulate the inflammatory response to enhance peri-implant soft tissue healing

The inflammatory response plays a dual role in peri-implant soft tissue healing. A moderate inflammatory response can clear debris and initiate tissue regeneration, while excessive or prolonged inflammation may hinder healing and implant stability. Effective inflammation regulation creates an optimal healing environment to promote recovery. Below, we discuss the mechanisms by which Mg-containing coatings regulate inflammation.

### 5.1 Regulation of immune cell behavior

**Figure 4:**
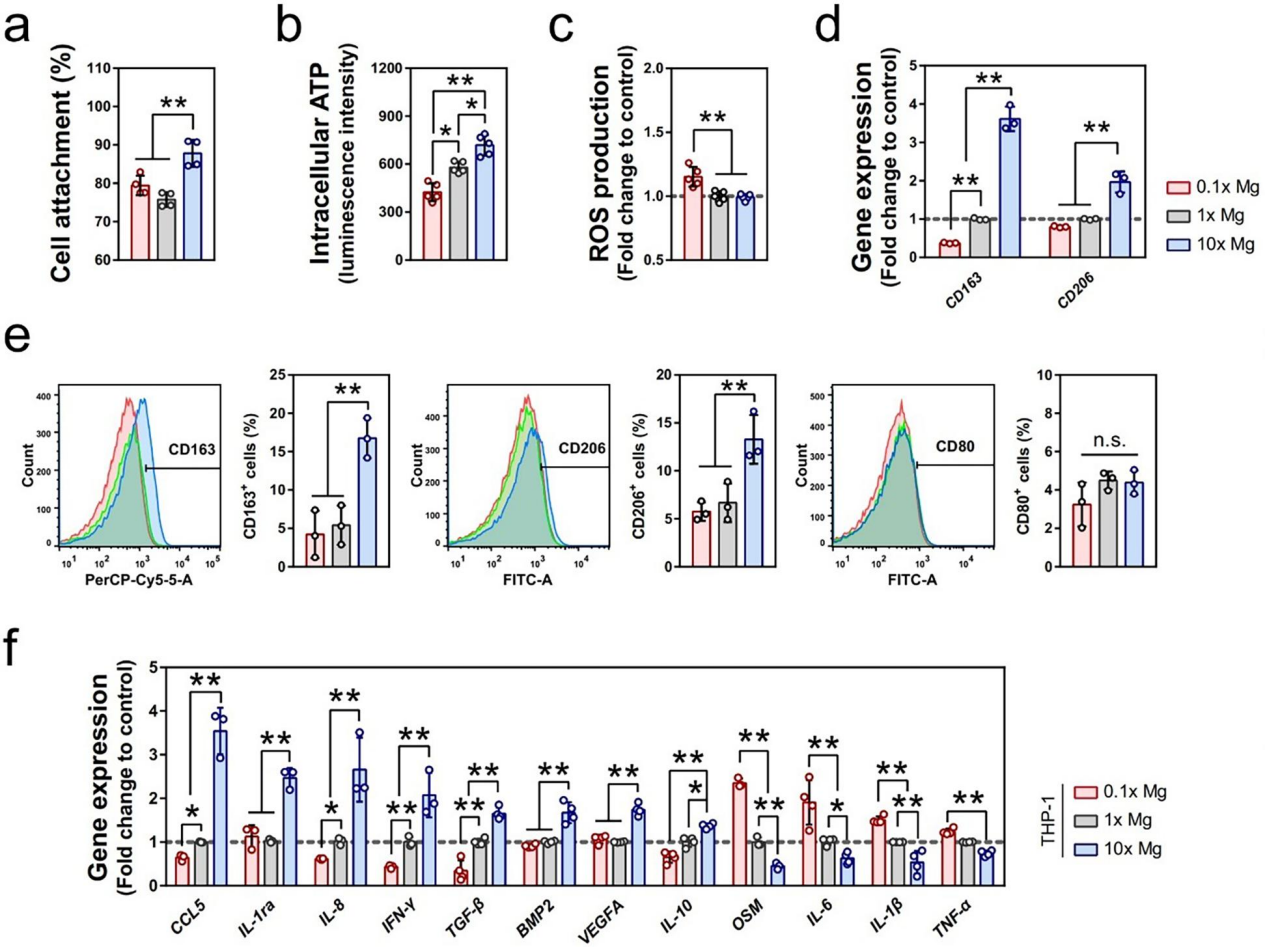
Mg^2+^ regulated the inflammatory microenvironment through the immunomodulation of macrophages. **(a–c)** The effects of different concentrations of Mg^2+^ on the cell attachment (a, *n* = 4), intracellular ATP level (b, *n* = 5), and ROS production (c, *n* = 4) of macrophages differentiated from suspension THP1 monocytes. The data for cell attachment was expressed as a percentage of initially seeded THP-1 cells. **(d)** The effect of different concentrations of Mg^2+^ on the gene expression of CD163 and CD206 in THP1-derived macrophages as evaluated by RT-qPCR (*n* = 3). **(e)** The effect of different concentrations of Mg^2+^ on the polarization of macrophages was evaluated by the expression of CD163, CD206, and CD80 using flow cytometry (*n* = 3). **(f)** The relative expression of inflammatory-related genes regulated by the stimulation of Mg^2+^ in THP1-derived macrophages (*n* = 3). Cited from Qiao W et al. (53) under the terms of the Creative Commons Attribution International License (CC BY4.0).

Mg-containing coatings gradually degrade *in vivo* and release Mg^2+^. With an appropriate concentration, Mg^2+^ exhibits significant anti-inflammatory properties, which is reflected in the regulation of immune cell behavior, especially macrophage polarization. Macrophages are categorized into pro-inflammatory M1 and anti-inflammatory M2 phenotypes based on their functions. During the acute inflammatory phase, M1 macrophages secrete cytokines like tumor necrosis factor (TNF)-*α* and IL-6 to eliminate pathogens and clear damaged tissue. However, a prolonged M1 response can lead to chronic inflammation and tissue damage (52). Separately, M2 macrophages promote tissue repair and regeneration by secreting anti-inflammatory cytokines such as IL-10 and TGF-β. Cerqueira et al. (50) successfully developed a sol-gel coating containing Mg and found that Mg2 + significantly reduced the level of TNF-α secreted by cells with an appropriate concentration while enhancing the expression of anti-inflammatory factors (TGF-β, IL-4). Qiao et al. (53) found via *in vitro* experiments in which THP1, a human monocyte line that can differentiate into macrophages, was exposed to different Mg^2+^ concentrations that increasing the Mg^2+^ concentration significantly promoted the maturation of THP1-derived macrophages from suspension monocytes to adherent macrophages and enhanced their activity. Gene-expression analysis showed that Mg^2+^ up-regulates M2 macrophage marker genes (such as *CD163* and *CD206*) and regulates osteoblast growth–related cytokine genes (such as *CCL5*, *IL-1ra*, and *TGF-β1*) while down-regulating osteoclast-promoting inflammatory factors (such as TNF-α and IL-1β). Cytokine array and Western blot analysis further confirmed that Mg^2+^ promotes the secretion of anti-inflammatory cytokines such as IL-1ra, IL-8, and CCL5 and inhibits the expression of IL-1β (as shown in Figure 4). Therefore, Mg-containing coatings can facilitate the shift from M1 to M2 macrophages through the release of Mg^2+^ (54), effectively reducing the duration of the inflammatory response and accelerating the healing process.

The specific effects of Mg^2+^ on macrophages have also been a recent research focus. Jin et al. (55) found that, under liposaccharide stimulation, Mg^2+^ can inhibit the TLR4–NF*κ*B pathway and promote anti-inflammatory cytokine secretion via activation of the TRPM7–PI3K–AKT pathway. Qiao et al. (53) further studied the role of TRPM7 in macrophages, showing that the regulatory effect of Mg^2+^ on pro-inflammatory cytokine secretion was significantly weakened when TRPM7 expression was inhibited using TRPM7 siRNA. This suggests that macrophages may sense and respond to Mg^2+^ through TRPM7.

In addition to macrophage polarization, Mg-containing coatings also regulate the behavior of other immune cells. Dendritic cells activate effector T-cells or regulatory T-cells via the uptake of antigens and cooperate with other immune cells such as macrophages to participate in tissue regeneration and barrier formation around the implant. Dai et al. (56) treated mice with bone defects with chitosan- and hyaluronic acid–coated Mg ion carriers and demonstrated that Mg^2+^ enters dendritic cells through the TRPM7 ion channel. The activation of the MAPK signaling pathway up-regulates the expression of hypoxia-inducible factor-1*α* and further promotes the secretion of TGF-β. Separately, TGF-β can inhibit the activation of effector T-cells (CD4^+^, CD8^+^) and increase the proportion of regulatory T-cells, thereby forming an immunosuppressive microenvironment and limiting the excessive immune response.

### 5.2 Regulation of the oxidative stress response

Oxidative stress is a key mechanism that initiates and sustains inflammation. During inflammation, immune cells like macrophages and neutrophils produce reactive oxygen species (ROS), which further amplify inflammation through pathways such as NADPH oxidase activation (57). However, excessive ROS can damage surrounding tissues and delay healing (58). Mg^2+^ effectively reduces oxidative stress levels by inhibiting ROS production, thereby alleviating tissue damage (57, 59). Mg^2+^ can also suppress inflammatory responses by regulating oxidative stress–related signaling pathways such as NF-*κ*B (60). NF-*κ*B is an important transcription factor that activates multiple genes associated with inflammation, so inhibiting the NF-*κ*B pathway can effectively reduce the release of pro-inflammatory cytokines. This mechanism not only reduces the initial inflammatory response but also helps prevent chronic inflammation in the long term.

Overall, Mg-containing coatings regulate the inflammatory response through multiple mechanisms. On the one hand, they achieve precise control of the inflammatory response by modulating immune cell behavior, particularly macrophage polarization; on the other hand, they reduce the intensity of inflammation by inhibiting oxidative stress. These anti-inflammatory and immunomodulatory effects demonstrate the broad potential of Mg-containing coatings in tissue repair and anti-inflammatory applications.

## 6 Mg-containing coatings enhance the antimicrobial properties of the abutment surface

In discussing the antimicrobial mechanisms of Mg-containing coatings, we analyze four primary mechanisms that contribute to their effectiveness and propose directions for future research.

### 6.1 Antibacterial ions release and pH modification

The primary antibacterial mechanism of Mg-containing coatings is the release of Mg^2+^. Higher concentrations of Mg^2+^ create greater osmotic pressure on bacteria, disrupting their physiological activities and inhibiting their growth and reproduction (61). The alkaline microenvironment created by Mg-containing coatings is another important factor contributing to their antibacterial properties. Most bacteria, especially anaerobes, struggle to survive and proliferate under high-pH conditions (62). This local alkaline environment not only inhibits bacterial growth but also prevents bacterial adhesion to the abutment surface, reducing biofilm formation (63). This is crucial for preventing postoperative infections and improving the long-term success rate of implants. The sustained ion release allows the coating to provide long-term antimicrobial protection for the abutment.

### 6.2 ROS generation

Mg^2+^ can promote the generation of ROS, especially hydroxyl radicals (•OH) and hydrogen peroxide (H_2_O_2_), through redox reactions (58). ROS can directly damage bacterial cell membranes and cause lipid peroxidation, ultimately resulting in bacterial death. Bacterial DNA can be oxidatively damaged by ROS, which will accelerate bacterial death. Also, ROS can oxidize proteins within cells, such as cysteine and methionine, causing protein denaturation or inactivation, which further inhibits bacterial growth and survival (64). The generation of ROS is a key mechanism in the antibacterial efficacy of Mg-containing coatings.

Tan et al. (65) used magnetron sputtering to deposit MgO film on biomedical titanium. Through their experiments, it was confirmed that MgO film would cause a lack of nutrients and ATP in bacteria, induce oxidative stress and death of said bacteria. This method only needs to increase the thickness of the MgO film to enhance its antibacterial effect. The specific mechanism is as follows: An appropriate transmembrane electrochemical proton gradient is necessary for the bacteria to pull extracellular H^+^ into the body by an F-type proton pump to produce ATP. Once the bacteria contact the MgO film, the extracellular H^+^ will be consumed and the proton gradient will be weakened, which will inhibit the synthesis of ATP and lead to a decrease in bacterial activity (65). The weakened proton gradient will interfere with the transmembrane transport of bacterial nutrients (66) and further affect bacterial metabolism. Oxygen is normally reduced to water by terminal oxidases in the respiratory electron transport chain that react on the bacterial membrane. However, due to the lack of protons in the alkaline microenvironment of bacteria, many oxygen molecules cannot obtain enough protons and eventually become ROS through the reaction. When the surface alkaline of MgO films is enhanced, bacteria will produce more ROS, which can damage bacterial enzymes, lipids and DNA, and other biomolecules, eventually leading to bacterial death. Although bacteria can regulate the acid–base balance inside and outside the cell through some ion channels, with the increase of MgO film thickness, the surface alkalinity rises beyond the threshold of the bacterial regulatory capacity, eventually leading to bacterial death.

### 6.3 Synergistic effects with other antibacterial materials

Mg-containing coatings can be combined with other antibacterial materials to enhance their antimicrobial efficacy. For instance, combining Mg with antimicrobial peptides (AMPs) has shown promising results. AMPs (67) primarily kill bacteria by disrupting bacterial cell membranes. A synergistic effect has been observed between Mg^2+^ and AMPs in the coating. Co-culture experiments with *Escherichia coli* (68) revealed that the coating maintained significant antibacterial efficacy even at low AMP concentrations, achieving a bactericidal rate of over 99%. Particularly, Mg oxide layers formed by electrodeposition combined with AMPs exhibited the most pronounced antibacterial effect. Kasi et al. (69) found that Mg-containing coatings combined with silver markedly enhanced antibacterial properties through multiple mechanisms, including metal ion release, ROS generation, and membrane disruption. This synergy also contributes to broad-spectrum antimicrobial activity against various microorganisms, such as bacteria and fungi. These composite coatings have demonstrated excellent antibacterial properties *in vitro* and in animal studies. In addition, research has explored the synergistic effects of Mg with other elements, such as iron (70) and Zn (71), to further enhance abutment coating antibacterial properties.

## 7 Challenges and future directions

Despite the promising effects of Mg-containing coatings in promoting soft tissue healing and providing antimicrobial properties, there are still several challenges in current research that require further exploration and optimization.

### 7.1 Ensuring long-term stability and controlled degradation

Controlling degradation kinetics and preventing cytotoxic Mg^2+^ bursts remain critical. Excessive Mg^2+^ concentrations may lead to dose-dependent cytotoxicity and genotoxicity (72), which could negatively impact the soft-tissue healing process. The oral microenvironment—characterized by dynamic masticatory forces, salivary flow, and pH fluctuations—accelerates coating degradation and complicates release predictability. While techniques like plasma immersion ion implantation enhance corrosion resistance, validation gaps persist, particularly in long-term (>6 months) *in vivo* models under oral-mimicking conditions. To bridge this, future studies must quantify real-time Mg^2+^ release profiles under mechanical stress and salivary exposure, establish epithelial/fibroblast viability thresholds, and comparatively evaluate degradation kinetics across key Mg compounds.

### 7.2 Advancing multifunctional composite coatings

Single Mg-containing coatings often struggle to simultaneously meet the requirements for antibacterial, corrosion-resistant, and bioactive properties. To address this, researchers are exploring multifunctional composite coatings by combining Mg with other materials. For instance, one study shows incorporating Mg and iron on a titanium substrate enhances fibroblast adhesion and promotes soft tissue healing (70). Similarly, co-implanting Mg^2+^ and Zn^2+^ ions into titanium substrates using plasma-implantation techniques has been shown to significantly promote the adhesion, migration, and proliferation of gingival fibroblasts (71). However, the clinical translation of such findings is hindered by undefined optimal formulations and species-specific dosing. Critically, current antimicrobial evaluations oversimplify oral ecology, focusing on single-pathogen models while neglecting complex polymicrobial biofilms. Future work should therefore prioritize three pillars: (1) screening broad-spectrum efficacy against multispecies biofilms, (2) quantifying epithelial sealing quality through hemidesmosome formation assays, and (3) systematically comparing adhesion strength across different multifunctional composite coatings.

### 7.3 Optimizing fabrication for clinical translation

Improving coating-fabrication techniques is another critical direction for future research. Currently, several methods for producing Mg-containing coatings, such as sol-gel, thermal spraying, and electrochemical deposition, face challenges, including technical complexity, high costs, and potential pollution. These issues limit the widespread clinical application of Mg-containing coatings. Therefore, next-generation techniques must concurrently achieve wear-resistant architectures able to sustain clinical function under masticatory stress for at least 5 years, low-energy consumption manufacturing processes, and eco-friendly deposition by eliminating toxic byproducts.

### 7.4 Bridging the translational Gap

The limited translatability of preclinical models necessitates prioritizing human clinical validation, starting with randomized controlled trials to assess (1) gingival seal integrity quantified via transmucosal resistance measurements and (2) 3–5-year peri-implantitis incidence rates. Concurrently, advanced *in vitro* models co-culturing human gingival fibroblasts with epithelial cells should be developed to predict mucosal responses. Crucially, clinical outcomes must be correlated with histological evidence of epithelial attachment integrity—particularly basal lamina continuity and hemidesmosome density—to resolve soft-tissue specificity gaps identified in preclinical studies.

## 8 Conclusion

**Figure 5:**
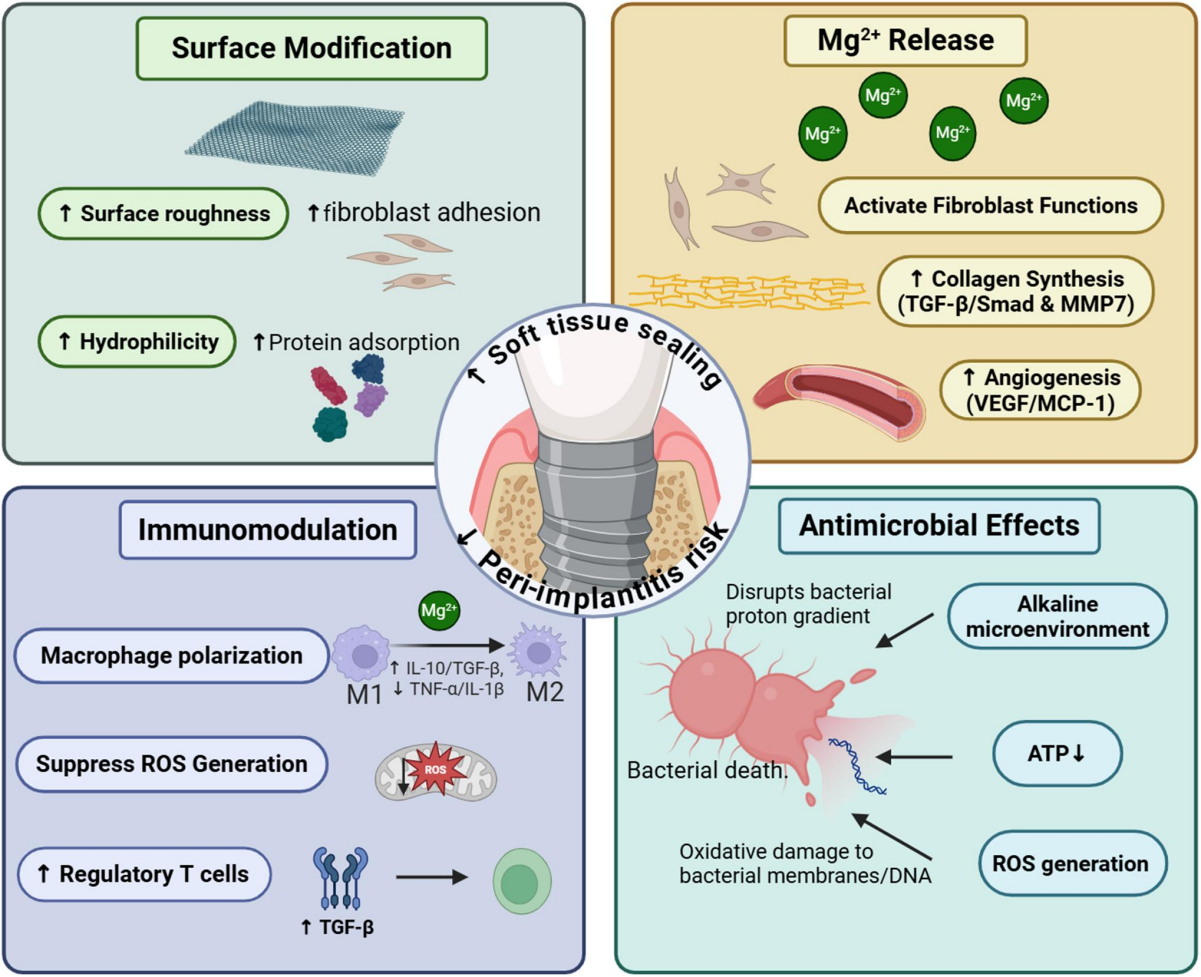
This figure summarizes the four cardinal functions of Mg-containing coatings in accelerating soft tissue healing around dental implants. This figure was created by the authors using licensed materials from https://BioRender.com.

Mg-containing coatings exhibit compelling preclinical potential for enhancing peri-implant soft tissue integration via multifaceted mechanisms, including surface modification promoting fibroblast/epithelial adhesion, Mg^2+^ release accelerating collagen synthesis and angiogenesis while modulating pro-healing macrophage polarization, and inherent antimicrobial activity combating biofilm formation,as shown in Figure 5. Critically, however, translational barriers impede clinical adoption. Key limitations include unresolved long-term coating stability under dynamic oral stresses, insufficient validation of epithelial sealing integrity (e.g., hemidesmosome formation at the abutment–mucosa interface), and variable bioactivity across Mg compounds. Future research must prioritize human randomized trials assessing mucosal seal durability and 3–5-year peri-implantitis rates, complemented by standardized degradation kinetics profiling under clinically relevant conditions. Concurrently, optimizing Mg formulation dosing and developing polymicrobial biofilm models will strengthen therapeutic generalizability. Resolving these gaps—particularly through histological confirmation of epithelial attachment structures and wear-resistant coating designs—will position Mg-coated abutments as transformative tools for preventing peri-implant complications and improving implant longevity.
